# The Influence of Episode Severity on Caregiver Recall, Care-seeking, and Treatment of Diarrhea among Children 2–59 Months of Age in Bihar, Gujarat, and Uttar Pradesh, India

**DOI:** 10.4269/ajtmh.14-0727

**Published:** 2015-08-05

**Authors:** Laura M. Lamberti, Christa L. Fischer Walker, Sunita Taneja, Sarmila Mazumder, Robert E. Black

**Affiliations:** Department of International Health, Johns Hopkins Bloomberg School of Public Health, Baltimore, Maryland; Center for Health Research and Development, Society for Applied Studies, New Delhi, India

## Abstract

Increased diarrheal episode severity has been linked to better 2-week recall and improved care-seeking and treatment among caregivers of children under five. Using cross-sectional data from three Indian states, we sought to assess the relationship between episode severity and the recall, care-seeking, and treatment of childhood diarrhea. Recall error was higher for episodes with onset 8–14 days (31.2%) versus 1–7 days (4.8%) before the survey, and logistic regression analysis showed a trend toward increased severity of less recent compared with more recent episodes. This finding indicates that data collection with 2-week recall underestimates diarrhea prevalence while overestimating the proportion of severe episodes. There was a strong correlation between care-seeking and dehydration, fever, vomiting, and increased stool frequency and duration. Treatment with oral rehydration salts was associated with dehydration, vomiting, and higher stool frequency, and trends were established between therapeutic zinc supplementation and increased duration and stool frequency. However, state and care-seeking sector were stronger determinants of treatment than episode severity, illustrating the need to address disparities in treatment quality across regions and delivery channels. Our findings are of importance to researchers and diarrhea management program evaluators aiming to produce accurate estimates of diarrheal outcomes and program impact in low- and middle-income countries.

## Background

Diarrhea is a leading cause of morbidity and mortality among children under five worldwide.^1^,^2^ Cross-sectional household surveys are typically used to measure diarrhea prevalence, care-seeking, and treatment among young children in low- and middle-income countries (LMIC). The advantages of cross-sectional data collection include estimation of point or period prevalence, as well as relative quickness and inexpensiveness when compared with longitudinal data collection.^3^ The 2-week period prevalence of diarrhea is a routinely collected indicator used to monitor the status of child health in countries worldwide.^4^,^5^

The accuracy of calling upon caregivers of children under five to recall the occurrence of diarrhea during the period of 2 weeks preceding a household visit has been called into question.^6^–^10^ Assuming that diarrhea is equally likely to occur at any point during a short time period (i.e., during the same diarrhea season), the number of episodes reported on each day within a recall interval should be comparable.^7^,^9^ However, studies have suggested that caregivers may fail to remember illness occurring earlier in the recall period, and such recall errors therefore bias estimates of 2-week diarrhea prevalence.^6^–^10^

Moreover, there is evidence that recall errors are more likely to occur for less severe diarrheal episodes among children under five^7^,^9^—a finding that supports the “Salient Principle,” which states that the accuracy of illness reporting improves when symptoms are more severe.^6^,^11^,^12^ A study in Bangladesh reported that diarrheal episodes accompanied by vomiting and higher stool frequency were reported more accurately,^9^ and a Guatemalan study concluded that in addition to underestimating the occurrence of diarrhea among children under five, longer recall periods disproportionately capture more severe episodes.^7^ Still, the “Salient Principle” has not been supported by other studies that used alternate definitions for severe diarrhea among children under five.^6^,^13^ Research is therefore warranted to establish the influence of diarrheal severity on caregiver recall in different geo-cultural contexts and utilizing varying definitions for what constitutes a severe episode.

In addition to influencing recall, studies in Yemen and India have suggested that perceptions about the severity of a child’s diarrheal episode may impact a caregiver’s decision to seek care outside the home and to purchase and/or administer certain treatments.^14^–^17^ A recent study used cross-sectional data from India to assess differentials in diarrhea care-seeking and treatment but noted lack of data on episode severity as a major limitation of the analysis.^18^

We sought to assess the influence of diarrheal episode severity on caregiver recall, care-seeking and adequate treatment (i.e., oral rehydration salts [ORS] and therapeutic zinc supplementation) for diarrhea among children under five using existing data collected for an external evaluation of two childhood diarrhea management programs in three Indian states.

## Methods

**Figure 1.**
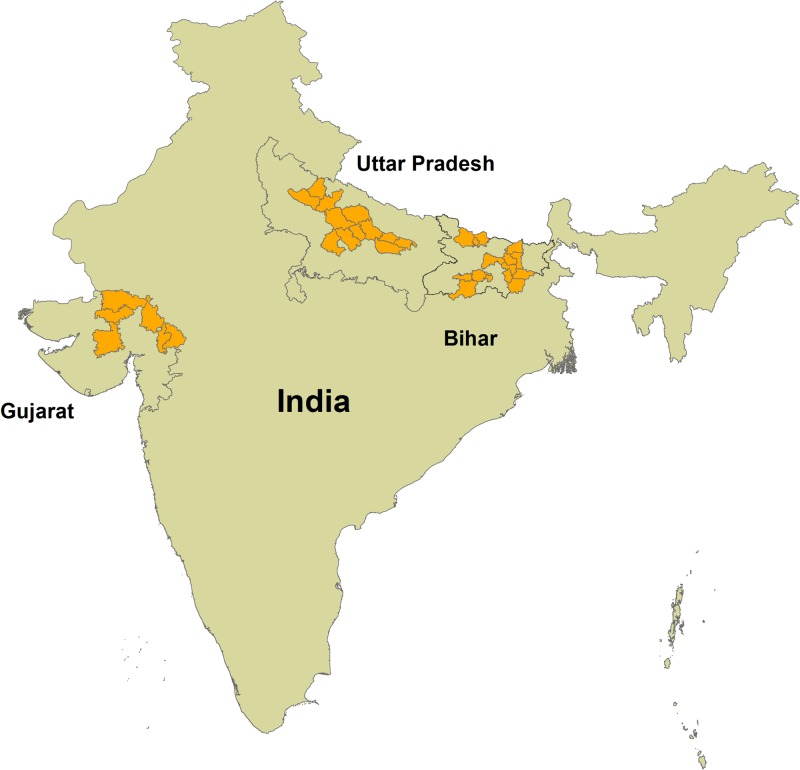
Districts included in data collection by state. *Source*: Map was generated using ArcGIS software and DIVA-GIS shapefiles.^19^,^20^ Twelve selected districts in UP (Ambedkar Nagar, Badaun, Bara Banki, Bareilly, Faizabad, Hardoi, Kanpur Dehat, Lucknow, Shahjahanpur, Sitapur, Sultanpur, and Unnao); 6 selected districts in Gujarat (Banas Kantha, Dohad, Panch Mahals, Patan, Sabar Kantha, and Surendranagar); 15 selected districts in Bihar (Banka, Bhagalpur, East Champaran, Gaya, Jehanabad, Khagaria, Madhepura, Munger, Nalanda, Saharsa, Samastipur, Sheikhpura, Sheohar, Sitamarhi, and Supaul).

In evaluating the Diarrhea Alleviation through Zinc and ORS Treatment program in Uttar Pradesh (UP) and Gujarat and a similar program in Bihar, we conducted multiple cross-sectional household surveys in program districts from March to June 2011 and September to October 2012 (Figure 1

). We divided the sample size required for each state equally across included districts and used a systematic sampling design to randomly select villages from each district. Data collectors enrolled a maximum of one caregiver of a child of 2–59 months of age per household. In households with multiple children of 2–59 months of age, the caregiver of the youngest child was invited to participate. Interviewers obtained informed consent before administering the survey. The first section of the survey included questions on demographics and diarrhea management knowledge, and the second section contained questions about diarrheal occurrence, care-seeking and treatment during the 2 weeks preceding the survey. In each village, data collection continued until either all households within the village were visited or the survey was administered to a maximum of 50 caregivers. Ethical approvals for all phases of the study were obtained from the Johns Hopkins University Institutional Review Board in Baltimore, MD and from the Society for Applied Studies Ethical Review Committee in New Delhi, India.

To define indicators of diarrheal severity, we used caregivers' responses to survey questions on signs and symptoms during the episode. We generated binary indicators using caregiver report of the presence or absence of blood in stools, fever, and vomiting during the diarrheal episode. We defined a discrete variable for reported maximum stool frequency, as well as a binary indicator of whether stool frequency exceeded five stools per day. For episodes that had resolved before the time of the survey, we defined a discrete variable for the duration of the diarrheal episode in days using the difference between the reported dates of episode recovery and episode onset; for episodes still in progress at the time of the survey, we defined duration as the difference between the date of the survey and the reported date of diarrhea onset. By these definitions, there were four extreme values for diarrheal duration, which were identified by plotting the studentized residuals and Cook's distances, and the extreme values were subsequently dropped from the analysis under the assumption that either onset dates had been erroneously reported or illness was chronic and therefore not comparable to the other episodes in the data set. Using the World Health Organization Integrated Management of Childhood Illness (IMCI) classification criteria for diarrhea-associated dehydration, we defined a binary variable for “any dehydration” (i.e., some or severe dehydration) based on available data^21^; “any dehydration” was therefore defined as two or more of the following signs: lethargy/irritability; sunken eyes; the inability to drink, or drinking poorly or extreme thirst.^21^

We based our analysis of the influence of diarrheal severity on caregiver recall on the assumption that diarrheal episodes occur with uniform distribution over a given recall interval and that other factors account for the skewed distribution of recalled episodes by onset date, which are generally underreported at > 1–2 days before data collection.^7^,^9^ To gauge whether episode recall waned over time, we calculated recall errors for the periods 1–7 and 8–14 days before the survey. Recall error is a measure of the percentage difference between the number of episodes with reported onset during a given interval and the number that would have been reported if the reporting rate had been consistent with that for episodes with onset 1–2 days before the survey.^7^,^9^ We used the following formula to calculate the recall error for the period 1–7 days before the survey^7^,^9^:

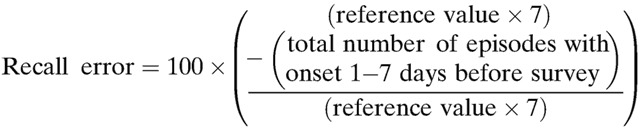


Reference value refers to the average number of episodes with reported onset 1–2 days before the survey (i.e., the total number of episodes with onset 1–2 days before the survey divided by 2). We calculated the recall error for the period 8–14 days before the survey by substituting the total number of episodes with onset 8–14 days before the survey into the numerator of the formula. Episodes occurring on the date of data collection were not included in this calculation because they were not representative of a full day of data collection (*N* = 4).

We also built logistic regression models to assess the influence of diarrheal episode severity on caregiver recall and restricted these analyses to data from children who had recovered from diarrhea by the time of the survey and whose episode onset did not exceed 14 days before the survey. We conducted bivariate and multivariable logistic regression analyses to determine whether the odds of various indicators of diarrheal severity were elevated among episodes with less recent onset (i.e., 8–14 days before the survey) compared with those with more recent onset (i.e., 3–7 days before the survey).

To determine whether the odds of care-seeking and treatment with ORS/zinc were higher comparing children with more severe and less severe episodes, we used data from all children with an episode in the 2 weeks preceding the survey and generated three binary outcome variables: care sought outside the home; receipt of ORS treatment; receipt of zinc treatment. We subsequently regressed each outcome onto the aforementioned indicators of diarrheal severity. Using data from those who sought care outside the home, we built a multinomial logistic regression model to assess the association between diarrheal severity and seeking care through either the public sector alone, the private sector alone, or both sectors. Sources of public sector care-seeking included primary health centers, auxiliary nurse midwives, Anganwadi workers, and Accredited Social Health Activists; and sources of private sector care-seeking included private doctors and hospitals, chemists, traditional healers, and unqualified private providers.

We conducted all logistic regression analyses in Stata 12.0 (Stata Corp., College Station, TX) employing the robust cluster estimator of variance with village defined as the cluster variable.^22^ We initially built separate models for each state, but the main effect sizes were comparable for Gujarat, Bihar, and UP; so we decided to combine the data and add a control variable for state of residence. All models also controlled for episode duration, child’s age and gender, years of caregiver education and phase of data collection (i.e., 2011 or 2012). In addition, the care-seeking and treatment models controlled for whether the child had recovered from the episode at the time of the survey, and the treatment models controlled for the interaction between state and care-seeking sector, because ORS/zinc products may not have been freely available through all sectors in every state. We inspected all models for statistically significant interactions between severity variables, child age and episode duration. We also used Wald tests of statistical significance and the Akaike Information Criterion to determine whether explanatory variables for severity and demographic variables should be retained in the final models.^23^

We collected data on 2,132 children with diarrhea in the 2 weeks preceding the survey, which granted adequate power to detect a range of reasonable effect sizes in the logistic regression analyses of care-seeking and treatment. Of the 2,132 children, 917 met the aforementioned criteria for inclusion in the logistic regression analysis of recall (i.e., episode recovered by the time of the survey and onset not exceeding 14 days before the survey), which also provided sufficient power.

## Results

### Demographic and diarrheal characteristics.

Table 1 outlines the key demographic and diarrheal episode characteristics of children with diarrhea in the 2 weeks preceding the survey. Commonly reported characteristics of diarrheal episodes included fever (72.3%) and vomiting (43.9%); whereas, maximum stool frequency > 5 stools/day (38.5%), any dehydration (25.4%) and blood in stools (12.2%) were less common. On average, maximum stool frequency was 5.6 stools per day (standard deviation [SD]: 2.5) and mean episode duration was 4.4 days (SD: 3.9). At the time of the survey, less than half of included episodes (44%) met the definition for a resolved episode (i.e., at least 72 hours without passing a loose/watery stool).

### The influence of diarrheal severity on caregiver recall of diarrhea.

**Figure 2.**
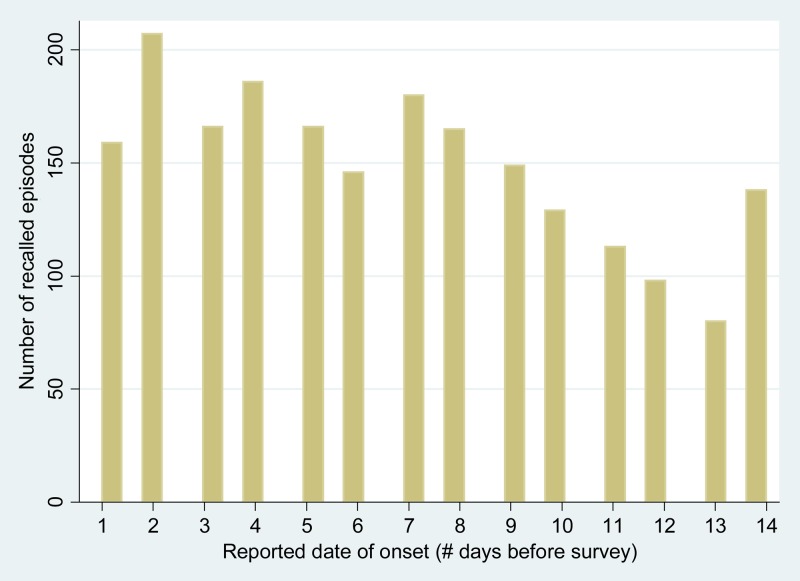
Distribution of recalled diarrheal episodes (*N* = 2,132 episodes) by reported date of onset (episodes with reported onset on the day of the survey were combined with those starting 1 day before the survey, since the survey date was not a full day of observation).

The recall errors for the periods of 1–7 and 8–14 days before the survey were 4.8% and 31.2%, respectively. Figure 2

illustrates that the distribution of all 2,132 recalled diarrheal episodes by reported date of onset was not uniform across the period of time preceding the survey. The large peak at 2 days indicates that reported onset of recalled episodes most commonly occurred 2 days before data collection. Following 2 days, the number of recalled episodes declined over time with a slight peak at 7 days and a larger peak at 14 days.

Of the 917 children included in the regression analysis, the reported date of diarrhea onset occurred 3–7 days before the survey for 284 (31.0%) and 8–14 days before the survey for 633 (69.0%). In bivariate analyses, the odds of severe diarrheal characteristics were statistically significantly elevated among children with less recent versus more recent diarrhea onset (Table 2). In multivariable analysis, there was a trend toward higher adjusted odds of any dehydration, blood in stools, fever and vomiting comparing episodes with onset 8–14 days before the survey to those with onset 3–7 days before the survey (Table 2). In addition, the odds of recalling a less recent episode with onset 8–14 days before the survey were elevated by 2.23 (95% CI: 1.79–2.79) per 1 day increase in episode duration.

### The influence of diarrheal characteristics on care-seeking.

**Figure 3.**
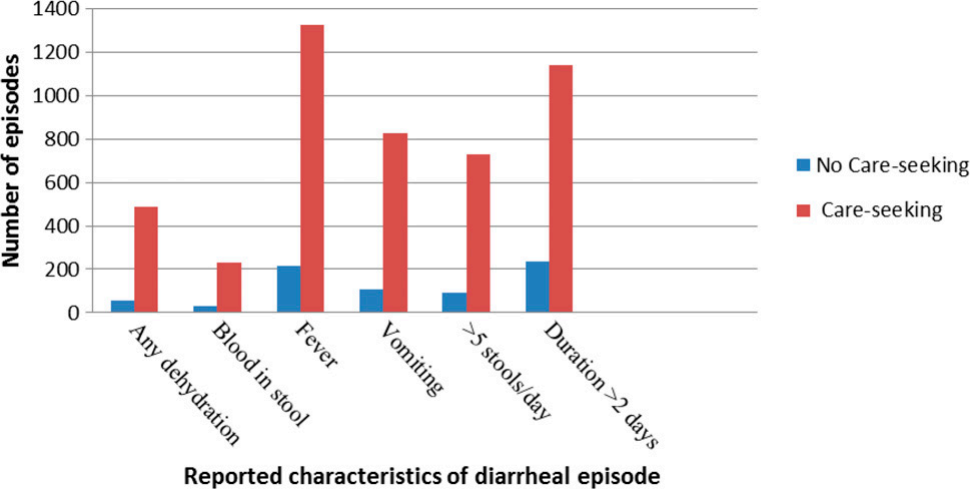
Trends in care-seeking among children with reported diarrheal characteristics.

Of the 2,132 caregivers included in this analysis, the majority (79.3%) reported having sought care outside the home for their child’s diarrheal episode (Table 1). Care was predominantly sought through the private sector alone (87.2%) compared with the public sector alone (5.8%) or to both sectors (3.7%) (Table 1). Figure 3

illustrates that the proportion of caregivers that sought care far exceeds the proportion that did not seek care for episodes with any dehydration, blood in stool, fever, vomiting, maximum stool frequency > 5 stools/day, and duration > 2 days. In multivariable logistic regression, the adjusted relative odds of care-seeking were elevated among children with any dehydration (adjusted odds ratio [aOR]: 1.73; 95% CI: 1.22–2.45), fever (aOR: 2.31; 95% CI: 1.79–2.98), vomiting (aOR: 1.93; 95% CI: 1.46–2.54), maximum stool frequency > 5 stools/day (aOR: 1.84; 95% CI: 1.36–2.48), and increased episode duration (aOR: 1.09; 95% CI: 1.03–1.16) (Table 3).

In the multinomial logistic regression model of care-seeking sector, the adjusted relative risk of seeking care through the private sector alone versus the public sector alone was 1.79 (95% CI: 1.03–3.13) times higher among children with any dehydration (Table 4). In addition, the relative risk of seeking care through both sectors as compared with the private sector alone increased by 15% (95% CI: 6–25%) per 1 stool/day increase in maximum stool frequency, controlling for other variables (Table 4). The relative risk of public versus private sector care-seeking was elevated by a factor of: 6.26 (95% CI: 3.51–11.16) in Gujarat compared with UP, 1.27 (95% CI: 0.57–2.81) in Bihar compared with UP, and 4.93 (95% CI: 3.97–11.42) in Gujarat compared with Bihar (Table 4).

### The influence of diarrheal severity on treatment.

Treatment of diarrheal episodes with ORS (18.4%) and zinc (3.8%) was not commonly reported among caregivers (Table 1). The adjusted relative odds of ORS treatment were elevated among children with vomiting (aOR: 1.88; 95% CI: 1.45–2.43) and maximum stool frequency > 5 stools/day (aOR: 1.54; 95% CI: 1.21–1.97) (Table 5). There was a statistically significant interaction between the binary variable for any dehydration and the continuous variable for child age (*P* = 0.022); the adjusted relative odds of ORS treatment comparing dehydrated to nondehydrated children of 2 months of age was 1.64 (95% CI: 1.12–2.42), and this odds ratio (OR) decreased by 2.3% (95% CI: 0.3–4.2%) per 1 month increase in age, holding all other variables constant (Table 5). There was an interaction between the sector through which care was sought and state; in Gujarat, Bihar, and UP, the adjusted relative odds of receiving ORS treatment comparing any public sector care-seeking to private sector care-seeking alone were 4.67 (95% CI: 2.81–7.77), 4.38 (95% CI: 1.76–10.89), and 1.21 (95% CI: 0.55–2.69), respectively (Table 5).

Maximum stool frequency > 5 stools/day (aOR: 1.79; 95% CI: 1.08–2.95) and increased episode duration (aOR: 1.04; 95% CI: 0.98–1.10) were the only diarrheal characteristics illustrating a trend toward higher odds of zinc treatment (Table 5). The effect of care-seeking sector on zinc treatment was modified by state; the adjusted OR of receiving zinc treatment comparing any public sector care-seeking to private sector care-seeking alone, controlling for other factors affecting care-seeking, were 8.90 (95% CI: 3.35–23.64) in Gujarat, 21.06 (95% CI: 5.87–75.63) in Bihar, and 2.23 (95% CI: 0.59–8.38) in UP (Table 5).

## Discussion

The results of this study demonstrate the potential influence of diarrheal episode characteristics on the recall, care-seeking, and treatment of diarrhea among children under five. We found that caregiver recall of diarrhea among children under five wanes over time, as evidenced by increased recall error comparing the periods 1–7 and 8–14 days before the survey and by the nonuniform distribution of diarrheal episodes by onset date (Figure 2). Although onset was less commonly reported on days farther from the survey date, the odds of any dehydration, fever, vomiting, and higher stool frequency were elevated among children with less recent as opposed to more recent illness onset (Table 2). This finding illustrates that symptoms associated with more severe disease increased the likelihood that a more distant diarrheal episode was remembered and reported. Further research is warranted to determine whether caregivers better recall diarrheal episodes paired with these symptoms because they are considered indicative of increased diarrheal episode severity or because the symptoms themselves are more memorable.

There was a strong correlation between diarrhea care-seeking and any dehydration, fever, vomiting, and higher stool frequency (Table 3). In addition, any dehydration and vomiting were linked to receipt of ORS, and increased stool frequency was associated with both ORS and zinc treatment (Table 5). These results suggest that perceived episode severity influences a caregiver’s decision to seek care and treatment. It is also possible that caregivers are more likely to recall and report the symptoms of episodes for which care and treatment were obtained outside the home.

The IMCI guidelines were designed for use by health-care workers in a clinical setting,^21^ and therefore our application of these criteria to prompted survey questions on diarrheal episode characteristics may have overestimated the true prevalence of dehydration among diarrhea cases. Our measure of dehydration cannot be considered comparable to those reported by clinical studies. Despite these differences, our estimate of 25.4% dehydration is comparable to the 23% of mild, moderate, and severe diarrhea cases that exhibit any dehydration globally (23.0%).^24^

This study has implications for evaluations of programs aiming to improve the management of diarrhea among children under five in India. We found that cross-sectional data collection with 2-week recall leads to underestimation of 2-week diarrhea prevalence and overestimation of the proportion of diarrheal episodes that are severe. A recall interval of 1 week as opposed to 2 weeks would likely decrease recall errors and is therefore preferable for evaluations aiming to produce unbiased estimates of program impact on diarrhea prevalence over time; although it should be noted that the seasonality of diarrhea makes it inappropriate to assess change using 1- or 2-week period prevalence data unless it is done throughout the year or, less reliably, at two points in the same season. Moreover, given the level of recall bias and diarrhea seasonality, it is inappropriate to use such survey data to calculate annual incidence or prevalence of diarrhea as has been done for disease burden estimates.^25^ It should also be noted that decreasing the length of the recall interval will result in increased sample size requirements that may be logistically unfeasible, but evaluators should at the very least collect data on diarrhea onset at the 1- and 2-week marks to enable estimation of the level of recall decay present in the data.

Diarrhea management programs should also remain mindful of the influence of perceived episode severity on care-seeking and ORS/zinc treatment. Ideally, programs should encourage care-seeking and treatment of all diarrheal episodes among children under five, regardless of severity. However, further research is warranted to ascertain whether the promotion of care-seeking and treatment of perceivably less severe episodes has a measurable impact on under five mortality. It is possible that limited programmatic resources would be more effectively allocated to the promotion of care-seeking and treatment of episodes exhibiting certain symptoms and characteristics; future research should therefore outline the specific severity criteria that could be translated into simple promotional messages for caregivers of children under-five. This approach should also be evaluated to determine whether it achieves greater health impact than a nontargeted approach.

This analysis highlighted that the factors most associated with ORS and zinc treatment were not indicators of perceived severity, but rather state of residence and the sector through which care was sought. There was an interaction between state and care-seeking sector such that children treated in the public sector were more likely to have received ORS and zinc than those treated in the private sector in all three states but by different magnitudes (Table 5). This finding suggests that public sector diarrhea treatment is of better quality than that received through the private sector in all three states, but the discrepancy between the two sectors is larger in Gujarat and Bihar than in UP. The variable effect of public sector care-seeking on receipt of ORS/zinc by state is not unexpected, since oversight of public sector delivery channels, including procurement and distribution of ORS and zinc, occurs at a state level. Differentials in treatment between public and private sector delivery channels are concerning given that the private sector remains the leading source of care for diarrhea among children under five in all three states; moreover, in our data, dehydration was associated with private rather than public sector sources of care-seeking (Table 4). The aim of diarrhea management programs for young children in Gujarat, Bihar, and UP should therefore be 2-fold—to encourage public sector care-seeking for diarrhea by raising awareness that quality treatment is now available through public sector channels; and to improve treatment practices for childhood diarrhea through all commonly used sources, including often hard-to-reach informal private sector practitioners. Meeting these challenges will require strategies tailored to local, state-specific needs. In this vein, program evaluators must consider regional differences and set state-specific benchmarks for outcome and impact.

## Figures and Tables

**Table 1:** Reported demographic and diarrheal episode characteristics

	Bihar, *N* = 437, *n* (%)	Gujarat, *N* = 759, *n* (%)	UP, *N* = 936, *n* (%)	Total, *N* = 2132, *n* (%)
Demographic characteristics				
Male	252 (57.7)	399 (52.6)	481 (51.4)	1132 (53.1)
Child age				
Mean (SD) in months	17.8 (13.2)	16.9 (12.8)	19.1 (13.9)	18.0 (13.4)
2–11 months	190 (43.5)	336 (44.3)	351 (37.5)	877 (41.1)
12–23 months	124 (28.4)	216 (28.5)	299 (31.9)	639 (30.0)
≥ 24 months	123 (28.1)	207 (27.3)	286 (30.6)	616 (28.9)
Caregiver school attendance				
Mean (SD) in years	2.28 (3.7)	3.24 (4.08)	2.92 (4.2)	2.91 (4.1)
Any school (i.e., ≥ 1 year)	145 (33.2)	341 (44.9)	351 (37.5)	837 (39.3)
Diarrheal episode characteristics				
Any dehydration	128 (29.3)	136 (17.9)	278 (29.7)	542 (25.4)
Blood in stools	53 (12.1)	59 (7.8)	149 (15.9)	261 (12.2)
Fever	289 (66.1)	474 (62.5)	778 (83.1)	1541 (72.3)
Vomiting	185 (42.3)	267 (35.2)	484 (51.7)	936 (43.9)
Maximum stool frequency > 5 stools/day	160 (36.6)	168 (22.1)	493 (52.7)	821 (38.5)
Mean episode duration (SD) in days	4.23 (3.3)	3.84 (3.7)	4.92 (4.2)	4.40 (3.9)
Child recovered at time of survey*	184 (42.1)	369 (48.6)	385 (41.1)	938 (44.0)
Care-seeking and treatment				
Care-seeking outside the home	344 (78.7)	524 (69.0)	822 (87.8)	1690 (79.3)
Private sector only†	322 (93.6)	389 (78.7)	762 (95.7)	1473 (87.2)
Public sector only†	11 (3.2)	67 (13.6)	20 (2.5)	98 (5.8)
Public and private sectors†	11 (3.2)	38 (7.7)	14 (1.8)	63 (3.7)
Episode treated with ORS	86 (19.7)	116 (15.3)	190 (20.3)	392 (18.4)
Episode treated with zinc	16 (3.7)	28 (3.7)	37 (4.0)	81 (3.8)

* Children were considered recovered if loose/watery stools were not experienced for at least 72 hours.

† The number of caregivers seeking care outside the home was used as the denominator for percentage calculations. The sum of percentages < 100% because source of care-seeking was not specified for 56 (3.3%) care seekers.

**Table 2:** Factors associated with recall of less recent diarrheal episodes in bivariate and multivariable analyses*

	Unadjusted OR (95% CI)	Adjusted OR (95% CI)†
Any dehydration	1.59 (1.09–2.33)	1.54 (0.98–2.41)
Blood in stools	2.02 (1.11–3.70)	1.49 (0.78–2.85)
Fever	1.72 (1.18–2.50)	1.17 (0.77–1.77)
Vomiting	1.80 (1.29–2.53)	1.31 (0.89–1.93)
Maximum stool frequency		
> 5 stools/day	2.01 (1.34–3.02)	1.03 (0.65–1.63)
≤ 5 stools/day	1.0	1.0
Episode duration (days)	2.15 (1.79–2.59)	2.23 (1.79–2.79)
State		
Bihar	0.80 (1.79–2.59)	1.75 (1.03–2.98)
Gujarat	0.80 (0.63–1.63)	1.09 (0.75–1.58)
UP	1.0	1.0

CI = confidence interval; OR = odds ratio; UP = Uttar Pradesh.

* Outcome variable was defined as reported diarrhea onset 8–14 days before the survey (i.e., less recent) compared with 3–7 days before the survey (i.e., more recent). Logistic regression analyses were performed in Stata 12.0 with the robust cluster estimator of variance to account for intra-village correlation.^22^

† Analysis was adjusted for all listed variables, as well as child’s age and gender, caregiver education in total years of schooling, and phase of data collection.

**Table 3:** Factors associated with diarrhea care-seeking in bivariate and multivariable analyses*

	Unadjusted OR (95% CI)	Adjusted OR (95% CI)†
Any dehydration	2.72 (1.95–3.79)	1.73 (1.22–2.45)
Blood in stools	2.27 (1.55–3.31)	1.23 (0.82–1.86)
Fever	3.75 (2.99–4.71)	2.31 (1.79–2.98)
Vomiting	2.97 (2.35–3.76)	1.93 (1.46–2.54)
Maximum stool frequency		
> 5 stools/day	2.89 (2.24–3.71)	1.84 (1.36–2.48)
≤ 5 stools/day	1.0	1.0
Episode duration (days)	1.10 (1.05–1.15)	1.09 (1.03–1.16)
State		
UP	3.23 (2.43–4.30)	2.20 (1.59–3.03)
Bihar	1.66 (1.19–2.31)	1.75 (1.20–2.54)
Gujarat	1.0	1.0

CI = confidence interval; OR = odds ratio; UP = Uttar Pradesh.

* Logistic regression analyses were performed in Stata 12.0 with the robust cluster estimator of variance to account for intra-village correlation.^22^

† Analysis was adjusted for all listed variables, as well as child’s age and gender, caregiver education in total years of schooling, phase of data collection, and whether the child had recovered from the diarrheal episode at the time of the survey (i.e., did not pass loose/watery stools for ≥ 72 hours).

**Table 4:** Factors associated with where care was sought in multinomial regression analysis*

	Public sector only vs. private sector only Adjusted RRR (95% CI)*	Both sectors vs. private sector only Adjusted RRR (95% CI)*
Any dehydration	0.56 (0.32–0.97)	0.87 (0.47–1.61)
Maximum stool frequency (stools/day)	1.02 (0.92–1.13)	1.15 (1.06–1.25)
Episode duration (days)	0.96 (0.88–1.05)	1.03 (0.95–1.11)
State		
Gujarat	6.26 (3.51–11.16)	7.12 (3.39–14.97)
Bihar	1.27 (0.57–2.81)	2.16 (0.89–5.27)
UP	1.0	1.0

CI = confidence interval; UP = Uttar Pradesh; RRR = relative risk ratio.

* Multinomial logistic regression analysis was performed in Stata 12.0 to model the categorical dependent outcome variable of where care was sought: 0 = private sector only; 1 = public sector only; 2 = both sectors. The robust cluster estimator of variance was used to account for intra-village correlation.^22^ Analysis was adjusted for all listed variables, as well as child’s age and gender, caregiver education in total years of schooling, phase of data collection, and whether the child had recovered from the diarrheal episode at the time of the survey (i.e., did not pass loose/watery stools for ≥ 72 hours). Fever, vomiting and blood in stools were not associated with care-seeking channel and thus not retained in the final model.

**Table 5:** Factors associated with ORS and zinc treatment of diarrhea in bivariate and multivariable analyses*

	ORS		Zinc	
	Unadjusted OR (95% CI)	Adjusted OR (95% CI)†	Unadjusted OR (95% CI)	Adjusted OR (95% CI)†
Any dehydration	1.49 (1.18–1.87)	1.64 (1.12–2.42)‡	1.02 (0.62–1.69)	1.02 (0.60–1.74)
Blood in stools	1.09 (0.79–1.50)	0.87 (0.62–1.22)	0.89 (0.44–1.79)	0.81 (0.41–1.58)
Fever	1.59 (1.20–2.10)	0.95 (0.69–1.31)	1.36 (0.80–2.29)	0.90 (0.49–1.64)
Vomiting	2.34 (1.84–2.96)	1.88 (1.45–2.43)	1.08 (0.69–1.69)	0.94 (0.58–1.54)
Maximum stool frequency				
> 5 stools/day	1.90 (1.54–2.35)	1.54 (1.21–1.97)	1.76 (1.13–2.73)	1.79 (1.08–2.95)
≤ 5 stools/day	1.0	1.0	1.0	1.0
Episode duration (days)	1.01 (0.99–1.04)	0.99 (0.95–1.02)	1.02 (0.98–1.07)	1.04 (0.98–1.10)
Care sought through public vs. private sector§				
Gujarat	4.53 (2.81–7.28)	4.67 (2.81–7.77)	8.74 (3.35–22.80)	8.90 (3.35–23.64)
Bihar	4.17 (1.72–10.12)	4.38 (1.76–10.89)	18.3 (5.19–64.59)	21.06 (5.87–75.63)
UP	1.18 (0.58–2.42)	1.21 (0.55–2.69)	2.21 (0.65–7.48)	2.23 (0.59–8.38)

CI = confidence interval; OR = odds ratio; ORS = oral rehydration salts; UP = Uttar Pradesh.

* Logistic regression analyses were performed in Stata 12.0 with the robust cluster estimator of variance to account for intra-village correlation.^22^

† Analyses were adjusted for all listed variables, as well as child’s age and gender, caregiver education in total years of schooling, phase of data collection, and whether the child had recovered from the diarrheal episode at the time of the survey (i.e., did not pass loose/watery stools for ≥ 72 hours).

‡ There was a statistically significant interaction between the binary variable for any dehydration and the continuous variable for child age centered at 2 months (*P* = 0.022); the adjusted OR of ORS treatment among dehydrated children is 1.64 (95% CI: 1.12–2.42) at 2 months of age and decreases by 2.3% (95% CI: 0.3–4.2%) per one month increase in age, holding all other variables constant.

§ Analyses controlled for the interaction between indicator variables for state and the binary variable for where care was sought (1 = any public sector care-seeking; 0 = private sector only); for each state, effect sizes are reported as the odds of having received ORS/zinc if care was sought through any public sector channel as compared with the private sector.
